# Political regulations to advance health care system

**DOI:** 10.1186/1878-5085-5-S1-A2

**Published:** 2014-02-11

**Authors:** Olga Golubnitschaja

**Affiliations:** 1 Department of Radiology, Rheinische Friedrich-Wilhelms-University of Bonn, Germany

## 

Through the support of the Alexander von Humboldt Foundation (Prof. Dr. Lotta Salminen of Finland and the host supervisor Prof. Dr. Olga Golubnitschaja of Germany were awarded fellowship), in the years 2012–2013, EPMA has performed a specialised project focused on the collaboration among PPPM-related professional groups and policymakers. This project is aimed to identify problems and deficits in health care of common and pandemic chronic diseases such as type 2 Diabetes mellitus. Interviews performed with the experts from all countries of the European Union, Israel, Russia and Turkey resulted in the conclusion that one of the common deficits is missing communication and collaboration between health care professionals and decision-makers. Moreover, there are difficulties in accessing national regulatory bodies to enhance the message of patient-focused health care. This evidence demonstrates that decisions in the health care sector are generally made without consideration of accumulated professional knowledge. This may explain the lack of sufficient efficacy currently observed in health care systems. The completed report with the collected data that resulted from this project is currently prepared for publication in *The EPMA Journal* (2014). In contrast, if well established, the collaboration with governmental institutions may lead to the crucial reconsideration of current national and European guidelines for health care relevant research activities and medical services. There are following emerging issues to be reconsidered in health care:

1. Promoting research fields focused on the patient needs

2. Mandating a paradigm shift from reactive to predictive and preventive medicine

3. Creating specialised state budgets to target preventive measures against pandemic incidence of common chronic diseases

4. Forcing an effective implementation of innovative predictive diagnostics

5. Creating new educational programmes for professionals to implement integrative medical services in predictive, preventive and personalised medicine

6. Creating educational measures to increase the understanding of innovative diagnostic technologies and targeted preventive measures in the general population

7. Creating new economic models to motivate health responsibility.

As health care is subject to national subsidiarity, there are substantial variations in the level of provision in different countries. However, corresponding standards might be introduced at the European level to promote inter-European mobility and to avoid economic discrepancies and conflict of interest [1,2].
